# A New Performance Measurement System for Maternal and Child Health in the United States

**DOI:** 10.1007/s10995-015-1739-5

**Published:** 2015-04-01

**Authors:** Michael D. Kogan, Christopher Dykton, Ashley H. Hirai, Bonnie B. Strickland, Christina D. Bethell, Iran Naqvi, Carlos E. Cano, Sheri L. Downing-Futrell, Michael C. Lu

**Affiliations:** 1 Maternal and Child Health Bureau, Health Resources and Services Administration, 5600 Fishers Lane, Rockville, MD 20857 USA; 2 Department of Family and Population Health, School of Public Health, Johns Hopkins University, Baltimore, MD USA

**Keywords:** Performance measurement, National performance measures, National outcome measures

## Abstract

**Objective:**

The Title V Maternal and Child Health (MCH) Block Grant is the linchpin for US MCH services. The first national performance measures (NPMs) for MCH were instituted in 1997. Changing trends in MCH risk factors, outcomes, health services, data sources, and advances in scientific knowledge, in conjunction with budgetary constraints led the Maternal and Child Health Bureau (MCHB) to design a new performance measurement system.

**Methods:**

A workgroup was formed to develop a new system. The following guiding principles were used: (1) Afford States more flexibility and reduce the overall reporting burden; (2) Improve accountability to better document Title V’s impact; (3) Develop NPMs that encompass measures in: maternal and women’s health, perinatal health, child health, children with special health care needs, adolescent health, and cross-cutting areas.

**Results:**

A three-tiered performance measurement system was proposed with national outcome measures (NOMs), NPMs and evidence-based/informed strategy measures (ESMs). NOMs are the ultimate goals that MCHB and States are attempting to achieve. NPMs are measures, generally associated with processes or programs, shown to affect NOMs. ESMs are evidence-based or informed measures that each State Title V program develops to affect the NPMs. There are 15 NPMs from which States select eight, with at least one from each population area. MCHB will provide the data for the NOMs and NPMs, when possible.

**Conclusions:**

The new performance measurement system increases the flexibility and reduces the reporting burden for States by allowing them to choose 8 NPMs to target, and increases accountability by having States develop actionable ESMs.

**Significance:**

The new national performance measure framework for maternal and child health will allow States more flexibility to address their areas of greatest need, reduce their data reporting burden by having the Maternal and Child Health Bureau provide data for the National Outcome and Performance Measures, yet afford States the opportunity to develop measurable strategies to address their selected performance measures.

## Introduction

The Title V Maternal and Child Health (MCH) Block Grant is the linchpin for MCH services in the United States. Administered by the Health Resources and Services Administration’s Maternal and Child Health Bureau (MCHB), the block grant operates through a Federal/State partnership in all 50 States, the District of Columbia and 9 jurisdictions. Title V was authorized in 1935 as part of the Social Security Act to stem the declining health of mothers and children in the midst of the Great Depression [1]. Title V became a block grant program as part of the Omnibus Budget Reconciliation Act (OBRA) of 1981 [2]. The Title V Block Grant was significantly modified through the OBRA of 1989, introducing greater accountability for the use of funds at both the Federal and State levels [3]. These standards for accountability were further strengthened in 1993 by The Government Performance Results Act (GPRA) [4]. The GPRA required program management tasks such as setting goals, measuring results, and reporting progress. In order to comply with GPRA, government agencies were required to prepare annual performance plans that established the performance goals for each fiscal year, and a description of how these goals were to be met.

As required by GPRA, the MCHB developed the first national performance measures (NPMs) for MCH in 1997, as part of a larger reporting system that included national outcome measures (NOMs), State performance measures, Health Systems Capacity Indicators, and Health Status Indicators. There were 18 NPMs, addressing issues such as newborn screening, breastfeeding, services for children with special health care needs, immunizations, the teen birth rate, deaths to children by motor vehicle crashes, hearing screening, children without health insurance, early prenatal care, obesity among children in the Women, Infants, and Children’s (WIC) program, and suicide deaths among teenagers (Table 1). These performance measures, among the first developed by a federal agency, were designed to address the most important issues facing MCH, and reflect the wide range of activities at the State level. There were also 6 NOMs: the infant mortality rate, the ratio of the black infant mortality rate to the white infant mortality rate, the neonatal mortality rate, the post-neonatal mortality rate, and the perinatal mortality rate. The NOMs were guided by legislation that mandated the collection of these data.

**Table 1:** Title V MCH Services Block Grant–previous national performance and outcome measures

No.	Measure
National performance measures	
1	The percent of screen positive newborns who received timely follow up to definitive diagnosis and clinical management for condition(s) mandated by their State-sponsored newborn screening programs
2	The percent of children with special health care needs age 0–18 years whose families partner in decision making at all levels and are satisfied with the services they receive (CSHCN survey)
3	The percent of children with special health care needs age 0–18 who receive coordinated, ongoing, comprehensive care within a medical home (CSHCN Survey)
4	The percent of children with special health care needs age 0–18 whose families have adequate private and/or public insurance to pay for the services they need (CSHCN Survey)
5	Percent of children with special health care needs age 0–18 whose families report the community-based service systems are organized so they can use them easily (CSHCN Survey)
6	The percentage of youth with special health care needs who received the services necessary to make transitions to all aspects of adult life, including adult health care, work, and independence
7	Percent of 19–35 month olds who have received full schedule of age appropriate immunizations against Measles, Mumps, Rubella, Polio, Diphtheria, Tetanus, Pertussis, Haemophilus Influenza, and Hepatitis B
8	The rate of birth (per 1000) for teenagers aged 15 through 17 years
9	Percent of third grade children who have received protective sealants on at least one permanent molar tooth
10	The rate of deaths to children aged 14 years and younger caused by motor vehicle crashes per 100,000 children
11	The percent of mothers who breastfeed their infants at 6 months of age
12	Percentage of newborns who have been screened for hearing before hospital discharge
13	Percent of children without health insurance
14	Percentage of children, ages 2–5 years, receiving WIC services with a Body Mass Index (BMI) at or above the 85th percentile
15	Percentage of women who smoke in the last 3 months of pregnancy
16	The rate (per 100,000) of suicide deaths among youths aged 15 through 19
17	Percent of very low birth weight infants delivered at facilities for high-risk deliveries and neonates
18	Percent of infants born to pregnant women receiving prenatal care beginning in the first trimester
National outcome measures	
1	The infant mortality rate per 1000 live births
2	The ratio of the black infant mortality rate to the white infant mortality rate
3	The neonatal mortality rate per 1000 live births
4	The postneonatal mortality rate per 1000 live births
5	The perinatal mortality rate per 1000 live births plus fetal deaths
6	The child death rate per 100,000 children aged 1 through 14

Concurrent with the development of the performance measures was the development of an information system in 1998, which became the Title V Information System (TVIS) [5, 6]. The TVIS is a conduit for electronic access to States’ reporting on the NPMs, NOMs, Health Status Indicators, Health Systems Capacity Indicators, financial data, and State narratives of related activities as a part of a required annual application and annual report.

Recently, the MCHB undertook the process of reassessing and revising the original Title V NPMs. Several trends underscored the need for this reexamination. First, numerous risk factors influencing MCH, as well as morbidity and mortality patterns, have changed in the last 15–20 years. Birth rates for teens aged 15–17 and 18–19 have fallen to record lows for both groups, declining over 50 % since their peak in 1991, although significant racial/ethnic disparities remain [7]. The preterm birth rate and low birth weight rates continued to rise until 2006, then began a slight decline, though they are still higher than they were during the 1990s [7]. Infant mortality declined through the 1990s, then remained generally stagnant until 2007, when multi-year declines resumed [8, 9], although the US infant mortality rate ranks 26th among industrialized countries [10]. The percent of children with chronic health conditions has increased greatly in the last two decades [11], with substantial increases in developmental and behavioral conditions such as autism spectrum disorder and attention deficit disorder [12–14]. Despite recent declines in obesity for young children aged 2–5 years, the obesity rate among children is still significantly higher today than in the early 1990s [15, 16].

Second, many changes have occurred in health services and health policy. The cesarean delivery rate increased about 60 % between 1996 and 2009, and has only dropped minimally since that time (32.9 % in 2009 to 32.7 % according to preliminary data) [7]. The percent of uninsured children declined from 13.9 % in 1997 to 6.5 % in 2013, in response to the introduction of the Children’s Health Insurance Program in 1997, expansions of Medicaid coverage, and the Affordable Care Act of 2010 [17, 18].

Third, there has been an increase in the amount and type of data available today compared to the 1990s, and increased awareness of the relevance of social determinants in MCH outcomes. The National Surveys of Children’s Health (NSCH), the National Surveys of Children with Special Health Care Needs (NS-CSHCN), and the American Community Survey did not exist at the time of the original performance measures. These surveys have provided a wealth of state-level data on the health and well-being of children, including information on children’s chronic health conditions, disability status, household poverty, social and emotional health, the family’s health, and the neighborhood environment. The 2003 revision to the U.S. Standard Certificate of Live Birth now provides critically new or improved detail on pre-pregnancy obesity, maternal smoking before and during pregnancy, birth spacing, maternal education, payment source, WIC receipt, breastfeeding at discharge, and various indicators of maternal-fetal morbidity, mode of delivery, and clinical care [19]. The Pregnancy Risk Assessment Monitoring System, which provides important information on maternal and infant health indicators before, during, and after pregnancy has also expanded its reach from 14 states in 1995 to 40 states currently [20].

Further, the measurement of performance and the science of MCH have advanced greatly since the 1990s. There has been a proliferation of measures related to MCH from Healthy People 20 [20, 21] the Children’s Health Insurance Program Reauthorization Acts’ (CHIPRA) Quality Improvement Measures [22], and the National Quality Forum [23], which has occurred in conjunction with advances in the science of performance measurement and the development of distinct methodologies for research, quality improvement and accountability purposes. Additionally, advances have occurred in the science of understanding the development of adverse health outcomes through the life course theory and the developmental origins of health and disease theory [24–27].

Finally, budgetary constraints in recent years have sharpened the need to establish the effectiveness of almost every government program. While each State reported annual goals, activities and accomplishments within the Title V Information System (TVIS), numerous factors reduced the ability to report this in aggregate to formulate a compelling national narrative on the work and accomplishments of this significant program. Beyond this, since the passage of the Affordable Care Act, it has become of utmost importance to explicitly measure the activities of Title V programs at both the Federal and State levels as they fill a unique function towards improving the health of the Nation’s mothers and children.

The purpose of this paper is to describe the development and rationale for a new Title V performance measure framework.

## Methods

A working group was formed as part of the visioning process to transform the Title V Block Grant program described elsewhere in this issue. The charge to the group was to assess the utility of the NPMs and NOMs, and, if necessary, develop a framework for a revised performance measure system. The work group began its deliberations by considering the major Title V investments in the States, the desired outcomes, logic models for different MCH programs, whether the existing performance measures could be affected by Title V programs, and whether there were reliable and timely national and State data sources for each measure.

After consideration, the work group determined that the present performance measurement system did not accurately reflect the impact of Title V program activities, and recommended that the original performance measures and NOMs be systematically reviewed and revised based on updated criteria. The work group based its recommendations on the finding that most existing performance measures were limited by one or more of the following data concerns:

- Limited availability of data, i.e. only once every 4 years for some measures.
- Dependence on data with questionable reliability.
- Lack of data standardization across the states, thereby limiting comparability.
- Structure and alignment of measures to Title V activities.
- Measures framed as performance measures which might be considered as outcome measures.
- Limited current relevance or importance of some performance measures.
- Limited relationship to the issues that represent the primary focus for Title V activities.
- Poor differentiation and some duplication between health status indicators and NPMs.
- NOMs focused only on mortality rather than reflecting the changing spectrum of children’s health conditions or the broader concept of children’s well-being.
- Performance measures that were not aligned with the NOMs.
- Limited opportunity for States to report on their measurable activities.

The work group believed decisions on NOMs and NPMs should be guided by a strategic framework which prioritized the identification of NPMs that could show the measurable impact of State Title V programs. Based upon the review of the original performance measures, the following guiding principles were adopted to facilitate the revision process.

1. Reduce the reporting burden for States. This decision was based on feedback from numerous States, reflecting the significant burden of reporting on 6 NOMs, 18 NPMs, 5 health status indicators, and 9 health systems capacity indicators. This frees States’ time and resources to focus on action toward improving the NPMs and using timelier, granular data to inform and evaluate state programmatic efforts.
2. Increase flexibility for States in choosing which performance measures to address. This decision recognized that States have very different needs and priorities. Expecting States to respond to a NPM that is not a State priority would be an inefficient use of time and/or funds.
3. Improve accountability and better document/monitor the impact of the Title V program. Measures should be actionable, quantifiable, and evidence-based or evidence-informed. The original performance measures required States to provide a narrative of their activities related to the performance measures. While a description of activities provides a wealth of information, in a time of increased competition for funds, as well as a greater policy and scientific focus on return on investment, it has become more urgent that States quantify and measure their activities related to the unique Title V functions, goals and priority outcomes.
4. Consider only those NPMs for which there are, or will be, reliable data sources. These data sources should be available annually, or, at most, every 2 years. Further, the data sources should provide both national estimates and State-level estimates for the majority of the States.
5. Select NPMs that encompass the diverse populations considered part of MCH. Such measures include the areas of maternal and women’s health, perinatal health, child health, adolescent and young adult health, children with special health care needs, and those that span multiple life stages.
6. Seek to enumerate performance measures considered to apply across life stages as the science behind the life course theory has grown significantly since the 1990s.
7. Stratify NPMs by risk groups, when possible, i.e. race/ethnicity, poverty status, level of urbanicity, and children with special health care needs status. This decision was borne by the data indicating that while some States may be doing well overall in a certain area, such as the percent of women who breastfeed their infants, there can be large disparities within the State that help identify areas for improvement and promote health equity—a HRSA strategic goal.
8. Determine that NPMs are modifiable. The work group made initial decisions as to whether a performance measure could be modified through the work of a State Title V program through careful review of existing activities in annual block grant reports, as well as more recent emerging issues and activities (e.g., perinatal quality collaboratives).
9. Create a framework where measurable activities at the State level could affect NPMs, and in turn, influence NOMs. The work group recognized a multitude of factors affecting changes in a performance measure. Many factors may be outside the purview of Title V, however, the work group believed it was important to bring more focus to activities of Title V that could be measured. For example, the percent of women who breastfeed their infants has been associated with sociodemographic factors, as well as the presence and type of breastfeeding legislation in a State [28, 29]. Breastfeeding initiation and duration may be positively influenced by delivery in Baby-Friendly Hospitals, particularly among women with lower education [30]. This is an area where Title V could play a role. However, breastfeeding practices are also associated with the need and plans to return to the workforce [31], an area where Title V may have less influence.

The work group developed criteria for a proposed three-tiered performance measure system: NOMs, NPMs, and evidence-based/informed strategy measures (ESMs).

Measures could be considered as NOMs, which are reflective of population health status, if they met one or more of the following criteria: it was mandated by the Title V legislation that the data be collected; it was considered a sentinel health marker for women, infants, or children; it was a major focus of either the Title V legislation or Title V activities; it was considered an important health condition to monitor because the prevalence was increasing, but the reasons for the increase were unclear; or there was a recognized need to move the MCH field forward in this area, even if there was not yet a consensus on how to measure the construct. The latter were considered developmental outcome measures.

Measures were examined for consideration as NPMs if they met one or more of the following criteria: there was a large investment of resources as determined by the State narratives; it was considered modifiable through Title V activities; a state could delineate measurable activities to address the performance measures; significant disparities existed among population groups; research had indicated that the condition or activity had large societal costs; or research had indicated that the promotion of certain behaviors, practices or policies had improved outcomes. There also had to be evidence that the performance measure was associated with at least one of the NOMs.

The ESMs are the key to understanding how a State Title V program tracks programmatic investments designed to impact the NPMs. In the framework, States create ESMs designed to impact NPMs. These measures would assess the impact of State Title V strategies and activities contained in the State Action Plan. It is envisioned that the development of ESMs will be guided through an examination of the evidenced-based or evidence-informed practices on what strategies and activities are both practical and measurable. The main criteria for ESMs would be that the activities had to be measurable, and there had to be evidence that the activity was related to the performance measure chosen. States could determine the number of ESMs that they would use for addressing the selected NPMs. States may also retire an ESM, if it has successfully achieved the NPM or a new ESM is introduced measuring a new, promising practice.

Over the course of a year, the work group, together with HRSA and MCHB leadership, developed a preliminary list of potential performance measures. Healthy People 2020, the CHIPRA health care quality measures, the National Quality Forum measures, the original Title V performance measures, and the performance measures developed by each State for Title V were among the sources examined.

The proposed NOMs and NPMs went through multiple iterations, often as a result of an intensive vetting process. The initial selection of NPMs was presented at the Association of Maternal and Child Health Programs Conference in January 2014. Stakeholders were encouraged to send comments by e-mail to MCHB. Almost 250 comments were received during this period. Numerous professional organizations were also consulted regarding specific NOMs and NPMs, including the American Academy of Pediatrics, the American College of Obstetricians and Gynecologists, the Association of Teachers of Maternal and Child Health, the American Academy of Pediatric Dentistry, and Family Voices. Other federal agencies such as the Centers for Disease Control and Prevention, and the Centers for Medicare and Medicaid Services were also consulted extensively. In the case of each measure, authorities on the subject and specialists in the field were invited to provide input in the creation and wording of the respective measures. There were often multiple measures to consider in each general domain (e.g., nutrition, oral health) and for discrete constructs (e.g., breastfeeding). This process allowed the most current science, existing or anticipated survey language and standard of care to be incorporated into the language of the measures. In June 2014, the proposed measures, as part of the draft of the Title V MCH Services Block Grant Guidance, were then open to comments from the public during the 60 days comment period that is a part of the approval process of the Office of Management and Budget (OMB). The revised Block Grant Guidance was open for a final 30 days public comment period in November 2014. The revised Block Grant guidance, including the performance measure framework, was approved by OMB in January 2015.

## Results

**Fig. 1:**
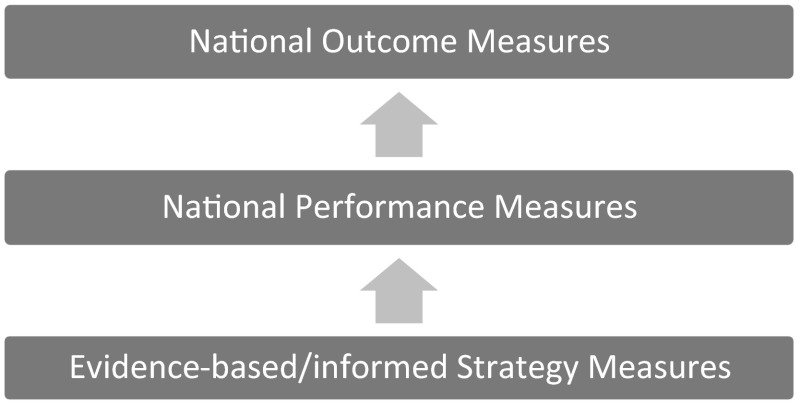
Title V MCH Services Block Grant–three-tiered performance measure framework

Figure 1 displays the three-tiered framework: NOMs, NPMs, and ESMs. NOMs are considered among the major goals MCHB and the States are attempting to achieve related to the health status of mothers and children. NPMs are measures, generally associated with processes or programs, which have been shown to affect the NOMs. However, it is important to recognize that associations may not be linear; there are many other influences to NOMs and movement in a given NPM may not necessarily result in movement of an NOM. ESMs are evidence-based/informed measures that each State Title V program develops to affect the NPMs.

The NOMs are shown in Table 2. There are now 22 NOMs, of which 11 are legislatively mandated, and generally reflect key indicators of morbidity and mortality. The other outcomes reflect commonly accepted indicators of a highly functioning system of care for children and their families: children having health insurance, a well-functioning system of care for children with special health care needs, and reduction of obesity among children; positive outcomes such as children in excellent or very good health and young children who are healthy and ready to learn (school readiness); outcomes which are a legislative focus for Title V such as the percent of children with special health care needs; outcomes reflecting the increase in developmental and behavioral conditions, such as autism spectrum disorders, attention deficit disorders, and treatment for children with mental or behavioral conditions; and developmental outcomes where either a fully functioning data system does not exist (e.g. newborn screening) or a consensus has yet to emerge on the best way to measure a concept (e.g. school readiness).

**Table 2:** Title V MCH Services Block Grant–national outcome measures

No.	Measure	Data source(s)
1	First trimester prenatal care entry (%)	National Vital Statistics System (NVSS)
2	Severe maternal morbidity per 10,000 deliveries	Healthcare Cost and Utilization Project-State Inpatient Database (HCUP-SID)
3	Maternal mortality rate per 100,000 live births	NVSS
4.1	Low birth weight deliveries (<2500 g) (%)	NVSS
4.2	Very low birth weight deliveries (<1500 g) (%)	NVSS
4.3	Moderately low birth weight deliveries (1500–2499 g) (%)	NVSS
5.1	Preterm births (<37 weeks’ gestation) (%)	NVSS
5.2	Early preterm births (<34 weeks’ gestation) (%)	NVSS
5.3	Late preterm births (34–36 weeks’ gestation) (%)	NVSS
6	Early term births (37, 38 weeks’ gestation) (%)	NVSS
7	Non-medically indicated early elective deliveries (37, 38 weeks’ gestation) (%)	Centers for Medicaid and Medicare Services (CMS) hospital compare
8	Perinatal mortality rate per 1000 live births plus fetal deaths	NVSS
9.1	Infant mortality rate per 1000 live births	NVSS
9.2	Neonatal mortality rate per 1000 live births	NVSS
9.3	Postneonatal mortality rate per 1000 live births	NVSS
9.4	Preterm-related mortality rate per 1000 live births	NVSS
9.5	Sudden Unexpected Infant Death (SUID) rate per 1000 live births	NVSS
10	Infants born with fetal alcohol exposure in the last 3 months of pregnancy (%)	Pregnancy risk assessment monitoring system
11	Neonatal abstinence syndrome per 1000 deliveries	HCUP-SID
12	Eligible newborns screened for heritable disorders with on time physician notification for out of range screens who are followed up in a timely manner (DEVELOPMENTAL) (%)	The American Public Health Laboratories data set
13	Children, ages 4–5, meeting the criteria developed for school readiness (DEVELOPMENTAL) (%)	National Survey of Children’s Health (NSCH)
14	Children, ages 1–17, who have decayed teeth or cavities in the past 12 months	NSCH
15	Child mortality rate ages 1 through 9 per 100,000	NVSS
16.1	Adolescent mortality rate ages 10 through 19 per 100,000	NVSS
16.2	Adolescent motor vehicle mortality rate ages 15 through 19 per 100,000	NVSS
16.3	Adolescent suicide rate ages 15 through 19 per 100,000	NVSS
17.1	Children with special health care needs (%)	NSCH
17.2	Children with special health care needs (CSHCN) receiving care in a well-functioning system (%)	NSCH
17.3	Children diagnosed with an autism spectrum disorder (%)	NSCH
17.4	Children diagnosed with Attention Deficit Disorder/Attention Deficit Hyperactivity Disorder (ADD/ADHD) (%)	NSCH
18	Children with a mental/behavioral condition who receive treatment (%)	NSCH
19	Children in excellent or very good health (%)	NSCH
20	Children and adolescents who are overweight or obese (BMI at or above the 85th percentile) (%)	WIC for children 2–4 years; NSCH for children 10–17 years (parent-report); YRBSS for adolescents grades 9–12
21	Children without health insurance (%)	American Community Survey
22.1	Children, ages 19–35 months, with the 4:3:1:3(4):3:1:4 combined series of vaccines (%)	National Immunization Survey (NIS)
22.2	Children, ages 6 months through 17 years, who are annually against seasonal influenza (%)	National Health Interview Survey (NHIS)
22.3	Adolescents, ages 13–17, who have received at least one dose of the HPV vaccine (%)	NIS
22.4	Adolescents, ages 13–17, who have received at least one dose of the Tdap vaccine (%)	NIS
22.5	Adolescents, ages 13–17, who have received at least one dose of the meningococcal conjugate vaccine (%)	NIS

There were 15 NPMs chosen (Table 3) covering six domains: women’s health, perinatal health, child health, adolescent and young adult health, children with special health care needs, as well as cross-cutting measures. Women’s health is represented by NPMs 1 and 2. NPM 1 is the percent of women with a past year preventive visit. The selection of this measure is consistent with MCHB’s overall focus on access to preventive health services, particularly given the emphasis in the Affordable Care Act surrounding women’s health preventive services and the importance of well-woman visits [32]. NPM 2, the percent of low-risk cesarean deliveries (37+ weeks, singleton, vertex births to nulliparous women), addresses the issue of maternal care quality given its connection to the most common causes of severe maternal morbidity (hemorrhage, infection, and embolism), as well as higher health care costs [33, 34]. Perinatal health is represented by NPMs 3, 4 and 5. National Performance Measure 3 is the percent of very low birth weight (VLBW) infants born in a hospital with a level III or higher Neonatal Intensive Care Unit (NICU). Although they represented less than 2 % of all births in 2010, VLBW infants accounted for 53 % of all infant deaths, with a risk of death over 100 times higher than that of normal birth weight infants (≥2500 g or 5.5 pounds) [35]. VLBW infants are significantly more likely to survive and thrive when born in a facility with a level-III NICU, a subspecialty facility equipped to handle high-risk neonates [36, 37]. NPM 4 is the percent of infants who were ever breastfed and the percent of infants breastfed through 6 months. While the breastfeeding initiation rate has continued to rise, large disparities still exist among states and racial/ethnic groups [28, 38]. Further, the Healthy People 2020 objective for breastfeeding at 6 months has not been attained yet [21]. NPM 5: the percent of mothers reporting they most often place their baby to sleep in a safe position reflects the fact that Sudden Unexpected Infant Deaths are still the leading cause of infant mortality after the first month of life, and the third leading cause overall [39]. National Performance Measure 6, focused on young children’s health and development, is the percent of children, 9 through 71 months old, who receive a developmental screening. The percent of children with developmental conditions is increasing, yet the percent of parents who reported that their child received developmental screening remains low [40]. NPMs 7 and 8 focus on both children’s and adolescents’ health. NPM 7: the percent of children admitted to a hospital with a diagnosis of unintentional or intentional injury, addresses the contribution of injuries towards child mortality and morbidity. NPM 8 is the percent of all children who are physically active 60 min a day consistent with the U.S. Department of Health and Human Services’ physical activity guidelines for all Americans aged 6 and older [41]. NPMs 9 and 10 are dedicated specifically to adolescent health: the percent of adolescents who are bullied (NPM 9) and the percent of adolescents with a preventive services visit in the last year (NPM 10). NPMs 11 (the percent of children with and without special health care needs having a medical home) and 12 (the percent of children with and without special health care needs who receive services necessary to make transitions to adult health care) are considered crucial to the development of a well-functioning system of care for children with special health care needs. NPMs 13, 14, and 15 could be considered as life course measures. NPM 13 is an integrated measure that addresses both the percent of women who had a dental visit during pregnancy and the percent of infants and children, ages 1–6 years, who had a preventive dental visit in the last year reflecting the importance of oral health throughout the life course. NPM 14 is also an integrated measure that focuses on the percent of women who smoke during pregnancy and the percent of children who live in households where someone smokes, highlighting the deleterious effects of smoking and tobacco exposure to both mothers and children. NPM 15 is the percent of children with adequate insurance. As more children are covered by health insurance, it will be vital that children are also adequately insured, especially certain groups such as children with special health care needs [42].

**Table 3:** Title V MCH Services Block Grant–national performance measures

No.	Measure	Data source(s)	MCH population domains
1	Percent of women with a past year preventive medical visit	Behavioral Risk Factor Surveillance System (BRFSS)	Women’s/maternal health
2	Percent of cesarean deliveries among low-risk first births	National Vital Statistics System (NVSS)	Women’s/maternal health
3	Percent of very low birth weight (VLBW) infants born in a hospital with a Level III+ Neonatal Intensive Care Unit (NICU)	Linked NVSS and American Academy of Pediatrics (AAP) data on hospital levels	Perinatal/infant health
4	(A) Percent of infants who are ever breastfed and (B) Percent of infants breastfed exclusively through 6 months	National Immunization Survey (NIS)	Perinatal/infant health
5	Percent of infants usually placed to sleep on their backs	Pregnancy Risk Assessment Monitoring System	Perinatal/infant health
6	Percent of children, ages 10–71 months, receiving a developmental screening using a parent-completed screening tool	National Survey of Children’s Health (NSCH)	Child health
7	Rate of hospitalization for non-fatal injury per 100,000 children ages 0–9 and adolescents ages 10–19	Healthcare Cost and Utilization Project—State inpatient database (HCUP-SID)	Child health and/or adolescent health
8	Percent of children ages 6–11 and adolescents ages 12–17 who are physically active at least 60 min per day	Youth Risk Behavior Surveillance System (YRBSS) and NSCH	Child health and/or adolescent health
9	Percent of adolescents, ages 12–17, who are bullied or who bully others	YRBSS and NSCH	Adolescent health
10	Percent of adolescents, ages 12–17, with a preventive medical visit in the past year	NSCH	Adolescent health
11	Percent of children with and without special health care needs having a medical home	NSCH	Children with special health care needs
12	Percent of children with and without special health care needs who received services necessary to make transitions to adult health care	NSCH	Children with special health care needs
13	(A) Percent of women who had a dental visit during pregnancy (B) Percent of infants and children, ages 1 through 17 years, who had a preventive dental visit in the past year	(A) PRAMS for dental visits during pregnancy and (B) NSCH for children’s visits	Cross-cutting/life course
14	(A) Percent of women who smoke during pregnancy (B) Percent of children who live in households where someone smokes	(A) NVSS for smoking during pregnancy and (B) NSCH for household smoking	Cross-cutting/life course
15	Percent of children 0–17 who are adequately insured	NSCH	Cross-cutting/life course

In keeping with MCHB’s plans to reduce burden and increase flexibility, States may choose 8 of the 15 NPMs, with at least one from each area. In addition, MCHB will provide the data for the NOMs and NPMs, when possible.

Table 4 displays all of the NOMs and NPMs, and indicates how they are associated. Table 5 provides a detailed example of how the framework might work using NPM 5 (percent of infants usually placed to sleep on their backs). The table indicates that it is associated with NOMs 9.1 (infant mortality rate), 9.3 (post-neonatal mortality rate), and 9.5 (Sudden Unexpected Infant Deaths). Examples of evidence-based/informed strategies, such as safe sleep protocols in birthing hospitals, are provided. An example of translating that strategy into an ESM follows. Using the same example, that would be: the percent of birthing hospitals that adopt safe sleep protocols. The final column then provides examples of including targets and goals into the ESM. Continuing with the same example would show: Increase the number of birthing hospitals in the State that adopt safe sleep protocols by 20 % in the next year. The sample strategies provided are drawn from the scientific literature [43–45].

**Table 4:** Performance measure framework: association between national performance measures and national outcome measures

	National performance measure (NPM)	National outcome measures associated with national performance measure
1	Well-woman visit (percent of women with a past year preventive medical visit)	Severe maternal morbidity per 10,000 delivery hospitalizations Maternal mortality rate per 100,000 live births Low birth weight rate (%) Very low birth weight rate (%) Moderately low birth weight rate (%) Preterm birth rate (%) Early preterm birth rate (%) Late preterm birth rate (%) Early term birth rate (%) Infant mortality per 1000 live births Perinatal mortality per 1000 live births plus fetal deaths Neonatal mortality per 1000 live births Postneonatal mortality rate per 1000 live births Preterm-related mortality per 100,000 live births
2	Low risk cesarean deliveries (percent of cesarean deliveries among low-risk first births)	Severe maternal morbidity per 10,000 delivery hospitalizations Maternal mortality rate per 100,000 live births
3	Perinatal regionalization [percent of very low birth weight (VLBW) infants born in a hospital with a Level III+ Neonatal Intensive Care Unit (NICU)]	Infant mortality per 1000 live births Perinatal mortality per 1000 live births plus fetal deaths Neonatal mortality per 1000 live births Preterm-related mortality per 100,000 live births
4	Breastfeeding (A. percent of infants who are ever breastfed and B. percent of infants breastfed exclusively through 6 months)	Infant mortality rate per 1000 live births Postneonatal mortality rate per 1000 live births Sleep-related SUID per 100,000 live births
5	Safe sleep (percent of infants placed to sleep on their backs)	Infant mortality per 1000 live births Post neonatal mortality per 1000 live births Sleep-related SUID per 100,000 live births
6	Developmental screening (percent of children, ages 10 through 71 months, receiving a developmental screening using a parent-completed screening tool)	Percent of children in excellent or very good health Percent of children meeting the criteria developed for school readiness
7	Child Injury (rate of hospitalization for non-fatal injury per 100,000 children ages 0 through 9 and adolescents ages 10 through 19)	Child mortality ages 1 through 9 per 100,000 Adolescent mortality ages 10 through 19 per 100,000 Adolescent motor vehicle mortality ages 15 through 19 per 100,000 Adolescent suicide ages 15 through 19 per 100,000
8	Physical activity (percent of children ages 6 through 11 and adolescents ages 12 through 17 who are physically active at least 60 min per day)	Percent of children in excellent or very good health Percent of children and adolescents who are overweight or obese (BMI at or above the 85th percentile)
9	Bullying (percent of adolescents, 12 through 17, who are bullied or who bully others)	Adolescent mortality ages 10 through 19 per 100,000 Adolescent suicide ages 15 through 19 per 100,000
10	Adolescent well-visit (percent of adolescents, ages 12 through 17, with a preventive medical visit in the past year)	Percent of children in excellent or very good health Percent of children ages 6 months through 17 years who are vaccinated annually against seasonal influenza Percent of adolescents, ages 13 through 17, who have received at least one dose of the HPV vaccine Percent of adolescents, ages 13 through 17, who have received at least one dose of the Tdap vaccine Percent of adolescents, ages 13 through 17, who have received at least one dose of the meningococcal conjugate vaccine Adolescent mortality ages 10 through 19 per 100,000 Adolescent motor vehicle mortality ages 15 through 19 per 100,000 Adolescent suicide ages 15 through 19 per 100,000 Percent of children with mental/behavioral health condition who receive treatment or counseling Percent of adolescents who are overweight or obese (BMI at or above the 85th percentile) Severe maternal morbidity per 10,000 delivery hospitalizations Maternal mortality rate per 100,000 live births Low birth weight rate (%) Very low birth weight rate (%) Moderately low birth weight rate (%) Preterm birth rate (%) Early preterm birth rate (%) Late preterm birth rate (%) Early term birth rate (%) Infant mortality per 1000 live births Perinatal mortality per 1000 live births plus fetal deaths Neonatal mortality per 1000 live births Postneonatal mortality rate per 1000 live births Preterm-related mortality per 100,000 live births
11	Medical home (percent of children with and without special health care needs having a medical home)	Percent of children with special health care needs (CSHCN) receiving care in a well-functioning system Percent of children in excellent or very good health Percent of children ages 19 through 35 months, who have received the 4:3:1:3(4):3:1:4 combined series of routine vaccinations Percent of children, ages 6 months through 17 years, who are vaccinated annually against seasonal influenza Percent of adolescents, ages 13 through 17, who have received at least one dose of the HPV vaccine Percent of adolescents, ages 13 through 17, who have received at least one dose of the Tdap vaccine Percent of adolescents, ages 13 through 17, who have received at least one dose of the meningococcal conjugate vaccine
12	Transition (percent of adolescents with and without special health care needs who received services necessary to make transitions to adult health care)	Percent of children with special health care needs (CSHCN) receiving care in a well-functioning system Percent of children in excellent or very good health
13	Oral health (A. percent of women who had a dental visit during pregnancy and B. percent of children, ages 1 through 17, who had a preventive dental visit in the past year)	Percent of children in excellent or very good health Percent of children ages 1 through 17 who have decayed teeth or cavities in the past 12 months
14	Smoking during pregnancy and household smoking (A. percent of women who smoke during pregnancy and B. percent of children who live in households where someone smokes)	Severe maternal morbidity per 10,000 delivery hospitalizations Maternal mortality rate per 100,000 live births Low birth weight rate (%) Very low birth weight rate (%) Moderately low birth weight rate (%) Preterm birth rate (%) Early preterm birth rate (%) Late preterm birth rate (%) Early term birth rate (%) Infant mortality per 1000 live births Perinatal mortality per 1000 live births plus fetal deaths Neonatal mortality per 1000 live births Preterm-related mortality per 100,000 live births Post neonatal mortality per 1000 live births Sleep-related SUID per 100,000 live births Percent of children in excellent or very good health
15	Adequate insurance coverage (percent of children ages 0 through 17 who are adequately insured)	Percent of children without health insurance Systems of care for children with special health care needs [percent of children and youth with special health care needs (CYSHCN) receiving care in a well-functioning system]

**Table 5:** Performance measure framework example using safe sleep: national outcome measures–national performance measures–evidence-based/informed strategy measures

National outcome measures associated with national performance measure on safe sleep	National performance measure (NPM)	Examples of evidence-based/informed strategies	Examples of evidence based/informed strategy measures	Examples of evidence based/informed strategy measures with targets and goals
Infant mortality per 1000 live births Post neonatal mortality per 1000 live births Sleep-related SUID mortality per 1000 live births	Safe sleep (percent of infants placed to sleep on their backs)	(a) Sudden Unexpected Infant Death (SUID) cases are reviewed by Child Death Review (CDR) teams using the CDC SUID Investigation Reporting Form and classification system (b) Analysis of Pregnancy Risk Assessment Monitoring System (PRAMS) and SUID-CDR data to identify program targets, inform interventions, develop fact sheets (c) Implementing a social marketing campaign (d) Partnership with the Womens, Infants and Children’s Program (WIC), Home Visiting, or other programs to provide safe sleep education and counseling (e) Safe sleep protocols in all birthing hospitals (f) Enforcing laws regarding mandatory training for childcare providers, medical professionals, emergency medical technicians (g) Train-the-trainer programs for the various providers engaged pre and post-natally	(a) % of SUID cases reviewed with complete SUID Investigation Reporting Forms and classified using CDC categories (b) # of state-wide or local programs integrating PRAMS/SUID data to develop or target interventions (c) # of hits to campaign website or hotline calls for more information (d) #/% of WIC participants. Home visiting clients, or other program participants that received safe sleep counseling (e) % of birthing hospitals that adopt safe sleep protocols (f) % of audited child care providers or other professionals in compliance with regulation (g) % of licensed medical professionals who received CE credits on SUID prevention or safe sleep practices in the past year	(a) Increase % of SUID cases reviewed with complete SUID Investigation Reporting Forms and classified using CDC categories to 90 % in the next year (b) Increase # of state-wide or local programs integrating PRAMS/SUID data to develop or target interventions by 20 % in the next year (c) Increase # of hits to campaign website or hotline calls for more information by 10 % in the next year the next year (d) Increase % of WIC participants, home visiting clients, or other program participants that received safe sleep counseling by 25 % in the next year (e) Increase # of birthing hospitals in the State that adopt safe sleep protocols by 20 % in the next year (f) Increase % of audited child care providers or other professionals in compliance with regulation by 35 % in the next year (g) Increase % of licensed medical professionals who received CE credits on SUID prevention or safe sleep practices by 20 % in the next year

## Discussion

The MCHB, in conjunction with multiple stakeholders, professional organizations, and partners set out to revise the Title V Performance Measures with the intention of reducing state reporting burden, and maintaining state flexibility while improving accountability of State Title V programs. A more integrated system has been created by developing a three-tiered performance measure framework that ties the ESMs to the NPMs that, in turn, may influence the NOMs. There will also be more depth in the system since data on the NPMs will be stratified by different risk factors, when available. First, burden has been reduced by having States work toward 8 NPMs rather than 18, and having MCHB supply the data for both NOMs and NPMs. Second, they can develop their own ESMs. Third, States will have the ability to change their ESMs, if they find they’re not achieving the desired results. There will be more accountability in the system due to the development of actionable measures by the States that can be tied to NPMs, which for the first time will be comparably/uniformly measured across states with the ability to aggregate to national levels.

However, reach of MCH is broad, and the selected NPMs account for only part of the myriad possible performance measures. MCHB considered many other PMs, such as preconception care, a postpartum visit, the percent of children living in safe neighborhoods, shared decision making between parents and providers regarding the child’s care, well-child visits in the first 15 months, screening for depression among adolescents, receipt of nutrition counseling for adolescents, use of tobacco or marijuana among adolescents, high school graduation, or fruit and vegetable consumption. Many possible measures were rejected because there was no reliable data source at both the national and state levels, while others were rejected because they were deemed outside the sphere of Title V’s influence. Further, even among selected NPMs, there were questions as to how best to frame the measure. For example, for the NPM on breastfeeding, the measure could have been initiation, duration, and/or exclusivity.

The creation of the Title V legislation has served as an essential element in propelling our Nation toward its goal of improving the health of women, children, and families. The partnership between the federal government and the States has enabled the development of best practices to ameliorate disparities and access issues for MCH populations. However, outcomes in MCH, both positive and negative, could be the result of one incident, like an accidental injury, or the culmination of generational choices and circumstances. The newly envisioned NOMs and NPMs are designed to reflect the breadth of factors that can influence health across the lifespan and/or have been shown to be mutable with the application of appropriate programmatic and policy levers. It is our hope that a more cohesive and comprehensive performance measurement system with a greater emphasis on measurable ESMs will be an important step that will maximize the impact of federal and State investments in MCH.

## References

[1] Understanding Title V of the Social Security Act (2002). Health Resources and Services Administration, Maternal and Child Health Bureau.

[2] Omnibus Budget Reconciliation Act of 1981. Public Law 97-35, 1981.

[3] Omnibus Budget Reconciliation Act of 1989. Public Law 101-239, 1989. 10113458

[4] Government Performance and Results Act of 1993. Public Law 103-62, 1993.

[5] Title V Maternal and Child Health Services Block Grant to the States Program: Guidance and Forms for the Title V Application/Annual Report (2012). Health Resources and Services Administration, Maternal and Child Health Bureau.

[6] https://mchdata.hrsa.gov/tvisreports/MeasurementData/MeasurementDataMenu.aspx. Last accessed on June 21, 2014.

[7] Hamilton, B. E., Martin, J. A., Osterman, M. J. K., Curtin, S. C. (2014). Births: Preliminary data for 2013. National vital statistics reports web release (Vol. 63, no. 02). Hyattsville, MD: National Center for Health Statistics. http://www.cdc.gov/nchs/data/nvsr/nvsr63/nvsr63_02.pdf. Last accessed on July 16, 2014.

[8] http://www.cdc.gov/nchs/data/dvs/2011_Final_Mortality_Data_Release.pdf. Last accessed on July 10, 2014.

[9] Murphy, S. L. (2000). Deaths: Final data for 1998. National vital statistics reports (Vol. 48, no. 11). Hyattsville, MD: National Center for Health Statistics. 10934859

[10] National Center for Health Statistics (2014). Health, United States, 2013: With special feature on prescription drugs. Hyattsville, Maryland. 24967476

[11] Perrin JM, Bloom SR, Gortmaker SL (2007). The increase of childhood chronic conditions in the United States. JAMA.

[12] Visser SN, Danielson ML, Bitsko RH, Holbrook JR, Kogan MD, Ghandour RM, Perou R, Blumberg SJ (2014). Trends in parent-report of health care provider diagnosed and medicated attention deficit/hyperactivity disorder in the United States, 2003–2011. Journal of the American Academy of Child and Adolescent Psychiatry.

[13] Boyle CA, Boulet S, Schieve LA, Cohen RA, Blumberg SJ, Yeargin-Allsopp M, Visser S, Kogan MD (2011). Trends in the prevalence of developmental disabilities in US children, 1997–2008. Pediatrics.

[14] Developmental Disabilities Monitoring Network Surveillance Year 2010 Principal Investigators; Centers for Disease Control and Prevention (CDC) (2014). Prevalence of autism spectrum disorder among children aged 8 years—Autism and developmental disabilities monitoring network, 11 sites, United States, 2010. *MMWR Surveillance Summaries 63*(2), 1–21. 24670961

[15] Ogden CL, Carroll MD, Kit BK, Flegal KM (2014). Prevalence of childhood and adult obesity in the United States, 2011–2012. JAMA.

[16] Lee H, Lee D, Guo G, Harris KM (2011). Trends in body mass index in adolescence and young adulthood in the United States, 1959–2002. Journal of Adolescent Health.

[17] Cohen, R. A., Martinez, M. E. (2014). Health insurance coverage: Early release of estimates from the National Health Interview Survey, 2013. National Center for Health Statistics. http://www.cdc.gov/nchs/nhis/releases.htm. Last accessed on June 21, 2014.

[18] Committee on Child Health Financing (2014). Children’s Health Insurance Program (CHIP): Accomplishments, challenges, and policy recommendations. Pediatrics.

[19] http://www.cdc.gov/nchs/nvss/vital_certificate_revisions.htm. Last accessed on July 10, 2014.

[20] http://www.cdc.gov/prams/statesyearsdata.htm#1998. Last accessed on July 10, 2014.

[21] http://www.healthypeople.gov/2020/default.aspx. Last accessed on January 26, 2015.

[22] http://www.medicaid.gov/Medicaid-CHIP-Program-Information/By-Topics/Quality-of-Care/CHIPRA-Initial-Core-Set-of-Childrens-Health-Care-Quality-Measures.html. Last accessed on June 29, 2014.

[23] http://www.qualityforum.org/Measuring_Performance/Measuring_Performance.aspx. Last accessed on June 29, 2014.

[24] Barker DJ (2007). The origins of the developmental origins theory. Journal of Internal Medicine.

[25] Lu MC, Halfon N (2003). Racial and ethnic disparities in birth outcomes: a life-course perspective. Maternal and Child Health Journal.

[26] Hertzman C (1999). The biological embedding of early experience and its effects on health in adulthood. Annals of the New York Academy of Sciences.

[27] Heckman JJ (2007). The economics, technology, and neuroscience of human capability formation. Proceedings of the national Academy of Sciences of USA.

[28] Kogan MD, Singh GK, Dee DL, Belanoff C, Grummer-Strawn LM (2008). Multivariate analysis of state variation in breastfeeding rates in the United States. American Journal of Public Health.

[29] Hawkins SS, Stern AD, Gillman MW (2013). Do state breastfeeding laws in the USA promote breast feeding?. Journal of Epidemiology and Community Health.

[30] Hawkins SS, Stern AD, Baum CF, Gillman MW (2014). Evaluating the impact of the Baby-Friendly Hospital initiative on breastfeeding rates: A multistate initiative. Public Health Nutrition.

[31] Mirkovic KR, Perrine CG, Scanlon KS, Grummer-Strawn LM (2014). In the United States, a mother’s plans for infant feeding are associated with her plans for employment. Journal of Human Lactation.

[32] http://www.hrsa.gov/womensguidelines. Last accessed on July 11, 2014.

[33] Main EK, Morton CH, Melsop K, Hopkins D, Giuliani G, Gould JB (2012). Creating a public agenda for maternity safety and quality in cesarean delivery. Obstetrics and Gynecology.

[34] American College of Obstetricians and Gynecologists (2014). Obstetric care consensus no. 1: Safe prevention of the primary cesarean delivery. Obstetrics & Gynecology.

[35] Mathews, T. J., MacDorman, M. F. (2013). Infant mortality statistics from the 2010 period linked birth/infant death data set. National vital statistics reports (Vol. 62, no. 8). Hyattsville, MD: National Center for Health Statistics. 24735562

[36] Lasswell SM, Barfield WD, Rochat RW, Blackmon L (2010). Perinatal regionalization for very low-birth-weight and very preterm infants: a meta-analysis. JAMA.

[37] American Academy of Pediatrics Committee on Fetus And Newborn (2012). Levels of neonatal care. Pediatrics.

[38] http://www.cdc.gov/BREASTFEEDING/DATA/NIS_data. Last accessed on July 11, 2014.

[39] Centers for Disease Control and Prevention, National Center for Health Statistics. Compressed mortality file 1999–2010 on CDC WONDER online database, released January 2013. Data are compiled from compressed mortality file 1999–2010 series 20 no. 2P, 2013. Last accessed at http://wonder.cdc.gov/cmf-icd10.html on July 10, 2014.

[40] http://childhealthdata.org/. Last accessed on June 30, 2014.

[41] http://www.nhlbi.nih.gov/health/health-topics/topics/phys/recommend.html. Last accessed on July 9, 2014.

[42] Kogan MD, Newacheck PW, Blumberg SJ, Ghandour RM, Singh GK, Strickland BB, van Dyck PC (2010). Underinsurance among children in the United States. New England Journal of Medicine.

[43] Moon RY, Oden RP, Grady KC (2004). Back to sleep: An educational intervention with women, infants, and children’s program clients. Pediatrics.

[44] Henderson-Smart DJ, Ponsonby AL, Murphy E (1998). Reducing the risk of sudden infant death syndrome: A review of the literature. Journal of Paediatrics and Child Health.

[45] Hitchcock SC, Owen KM, Young LJ (2013). Endorsing safe sleep: Helping nurses turn recommendations into reality. Journal of Obstetric, Gynecologic, and Neonatal Nursing.

